# REM sleep proportion: an independent predictor of olfactory dysfunction in obstructive sleep apnea hypopnea syndrome

**DOI:** 10.3389/fneur.2026.1815844

**Published:** 2026-05-27

**Authors:** Yuanquan Li, Renli Huang, Xingya Li, Qingchun Pan

**Affiliations:** 1 Affiliated Hospital of North Sichuan Medical College, Nanchong, Sichuan, China; 2 Longchang People’s Hospital, Longchang, Sichuan, China

**Keywords:** obstructive sleep apnea, olfactory dysfunction, REM sleep, risk factors, sleep architecture

## Abstract

**Objective:**

This study aimed to investigate the association between olfactory dysfunction and sleep architecture parameters in patients with obstructive sleep apnea-hypopnea syndrome (OSAHS), identify independent factors influencing olfactory function, and offer novel insights into the multisystemic impact of OSAHS.

**Methods:**

Employing a cross-sectional retrospective design, data from 198 patients diagnosed with OSAHS via polysomnography (PSG) from December 1, 2020, to June 30, 2024, were analyzed. Olfactory performance was assessed using the Sniffin’ Sticks test kit. Sleep structure parameters, including total sleep time (TST), sleep efficiency (SE), N3 sleep proportion (N3%), rapid eye movement sleep proportion (REM%), apnea-hypopnea index (AHI), and lowest oxygen saturation (LSaO2), were recorded by PSG. Pearson and Spearman correlation analyses were conducted to examine associations between olfactory scores and sleep parameters. Multivariate logistic regression was employed to determine independent predictors for olfactory impairment.

**Results:**

Male patients exhibited a significantly higher proportion in the olfactory dysfunction group (81/131) compared to the normal olfaction group (50/131; *p* < 0.05). Additionally, the olfactory impairment group showed significantly reduced total microarousals during REM and NREM sleep, N3/TST%, and REM/TST% compared to the normal olfaction group (*p* < 0.05). Conversely, AHI and wake after sleep onset (WASO) were notably increased in patients with olfactory impairment (*p* < 0.05). Olfactory threshold (T), discrimination (D), identification (I), and total TDI scores were significantly lower in patients with olfactory dysfunction (*p* < 0.05). Correlation analyses revealed negative correlations between total TDI scores and age, AHI, and WASO, while positive correlations were observed with TST, SE, total REM sleep microarousals, and REM/TST%. Logistic regression analysis further identified age, AHI, and REM/TST% as independent predictors influencing TDI scores.

**Conclusion:**

Age increase, elevated AHI and decreased REM/TST% are independent influencing factors for the occurrence of olfactory disorders. The decline in olfactory function is significantly associated with a marked reduction in REM sleep. Elderly patients with OSAHS, high AHI and low REM proportion should be the key population for olfactory function screening.

## 1 Introduction

Globally, obstructive sleep apnea-hypopnea syndrome (OSAHS), characterized by repeated collapse of the upper airway, remains one of the most prevalent respiratory sleep disorders. This condition results in intermittent episodes of reduced blood oxygen (hypoxia), elevated carbon dioxide levels (hypercapnia), and significant disruptions in the sleep structure (1). Recent epidemiological data indicate a prevalence of approximately 23.6% among Chinese adults, closely associated with multi-systemic complications, including metabolic syndrome, cardiovascular diseases, and cognitive impairment (2). Importantly, recent clinical evidence suggests that the prevalence of olfactory dysfunction in OSAHS patients (36.6–88.0%) significantly exceeds that in the general population and positively correlates with disease severity (3). Such impairment adversely impacts patient quality of life and may exacerbate cognitive decline via the olfactory-limbic system pathway, thereby highlighting olfactory dysfunction as a critical indicator of multisystemic involvement in OSAHS (4).

Current research on olfactory dysfunction in obstructive sleep apnea-hypopnea syndrome (OSAHS) mainly focuses on hypoxic injury mechanisms, suggesting that nasal airflow restriction and intermittent hypoxemia caused by upper airway obstruction are the main pathogenic factors (5). However, the integrity of the olfactory conduction pathway depends on the coordinated action of peripheral olfactory receptors and the central olfactory cortex, among which rapid eye movement (REM) sleep plays an irreplaceable role in the consolidation of olfactory memory (6). Animal experiments have shown that REM sleep deprivation can lead to weakened theta wave activity in the hippocampus and significantly reduce odor recognition memory ability. Clinical studies have also confirmed that the proportion of REM sleep is significantly positively correlated with human olfactory discrimination ability (3). Patients diagnosed with OSAHS commonly present notable alterations in sleep architecture. Specifically, these disturbances manifest as reductions in slow-wave sleep (stage N3) and fragmentation of rapid eye movement (REM) sleep. These abnormal sleep changes potentially affect the function of olfactory centers by influencing neural plasticity processes (4). Although prior studies indicate apnea-hypopnea index (AHI) and oxygen saturation as independent predictors of olfactory dysfunction, these conclusions largely stem from cross-sectional analyses lacking detailed evaluation of sleep parameters. More importantly, existing studies often simplify sleep structure into single indicators such as total sleep time or sleep efficiency, ignoring the functional heterogeneity of different sleep stages. In fact, REM sleep and NREM sleep have essential differences in neurotransmitter release and synaptic plasticity regulation, and their regulatory mechanisms on olfactory function may be completely different (7). Additionally, longitudinal studies on olfactory dysfunction in OSAHS patients are scarce, making it impossible to clarify the causal relationship between improvements in sleep structure parameters and the recovery of olfactory function.

This study innovatively integrates the Sniffin’ Sticks olfactory test with polysomnographic parameters, incorporating the proportion of REM sleep (REM/TST%) as an independent variable into the analysis system. Compared with traditional sleep efficiency or microarousal index, REM/TST% can better reflect the absolute amount and relative proportion of REM sleep, providing a more precise sleep structure indicator for evaluating olfactory function. Through cross-sectional data analysis, this study aims to reveal the independent role of REM sleep reduction in olfactory dysfunction in OSAHS, providing a new perspective for understanding its neural injury mechanism. At the same time, identifying the interaction effects of age, AHI, and REM/TST% can provide evidence-based basis for early screening and stratified management of high-risk populations, which has important clinical translational value.

## 2 Materials and methods

### 2.1 Research subjects

This study retrospectively enrolled 198 OSAHS patients diagnosed by polysomnography (PSG) between December 1, 2020, and June 30, 2024. A total of 114 patients were excluded: 42 due to nasal structural/pathological conditions (septal deviation, nasal polyps, chronic rhinosinusitis, atrophic rhinitis, acute allergic rhinitis), 31 due to neurodegenerative diseases (Alzheimer’s disease, Parkinson’s disease) or traumatic brain injury, 18 due to medication use affecting olfaction (glucocorticoids, antihistamines) within 1 month prior to enrollment, 13 due to incomplete PSG data, and 10 due to refusal to undergo olfactory testing. Finally, 198 patients were included. Inclusion criteria encompassed: ① Diagnostic criteria compliance per the International Classification of Sleep Disorders, Third Edition (ICSD-3), characterized by AHI ≥ 5 events/h predominantly due to obstructive apnea or hypopnea; ② Age ≥ 18 years; ③ Capability to perform olfactory function testing. Exclusion criteria included: ① History of nasal surgery, chronic sinusitis, nasal septum deviation, atrophic rhinitis, nasal polyps, or acute exacerbations of allergic rhinitis; ② Comorbid neurodegenerative conditions (e.g., Alzheimer’s or Parkinson’s disease) or history of traumatic brain injury; ③ Pregnancy or lactation; ④ Recent use (within 1 month before enrollment) of medications potentially affecting olfactory function (e.g., glucocorticoids, antihistamines). The cohort comprised 131 males and 67 females, with a mean age of 46 ± 12 years and mean education duration of 9 ± 3 years. Smoking status (pack-years), alcohol consumption, and diabetes history were additionally recorded as covariates.

### 2.2 Methods

#### 2.2.1 Polysomnography

Polysomnographic monitoring utilized the Somte E device (Compumedics, Australia), adhering to American Academy of Sleep Medicine (AASM) guidelines to record electroencephalogram (EEG), electrooculogram (EOG), submental electromyogram (EMG), nasal-oral airflow, thoraco-abdominal respiratory effort, blood oxygen saturation, and body position (8). Recorded sleep parameters included total sleep time (TST), sleep efficiency (SE), maximum duration of obstructive apnea (OA), minimum oxygen saturation, proportions of sleep stages (N1, N2, N3, and REM relative to total sleep time; N1/TST%, N2/TST%, N3/TST%, REM/TST%), AHI, total non-REM microarousals, REM-related microarousals, and wakefulness after sleep onset (WASO).

#### 2.2.2 Olfactory function assessment

The Sniffin’ Sticks olfactory detection kit (a standardized odor test tool) from Burghardt, Germany, was used for the assessment in an independent testing room with a temperature of (22 ± 1)°C and a humidity of (50 ± 5)%. The test was conducted by trained technicians. Threshold test (T): The odor perception threshold was detected using a concentration gradient of phenylethanol (1:2 dilution, 16 steps). When the patient correctly identified the target odor three consecutive times starting from the lowest concentration, the lowest concentration was recorded as the threshold score (0–16 points). Discrimination test (D): 16 groups of three-choice odor sticks (including 1 target odor and 2 interfering odors) were used, and the patient needed to identify the target odor. The discrimination (D) score (0–16 points) was determined by the number of correctly identified odors. Identification (I) testing involved 16 commonly encountered scents (e.g., coffee, mint), with correct responses selected from four provided options; the number of correct answers constituted the identification score (0–16 points). The overall TDI score was calculated as T + D + I. Reference ranges for olfactory function were: normal olfaction (TDI ≥ 31 points), mild impairment (28–30 points), moderate impairment (16–27 points), and severe impairment (≤15 points) (9). Patients with TDI ≤ 30 points were categorized into the olfactory impairment group, whereas those scoring above 30 points were considered to have normal olfactory function.

### 2.3 Statistical analysis

Statistical analyses in this study utilized SPSS software version 26.0. Continuous data underwent normality testing via the Shapiro–Wilk test. Variables following a normal distribution were expressed using mean ± standard deviation (SD) and analyzed by independent-sample t-tests. Variables not conforming to normal distribution were described using median and interquartile range (P25, P75) and analyzed through the Mann–Whitney *U* test. Categorical data were presented as frequency distributions and assessed by chi-square testing. Initially, univariate analyses were applied to demographic and polysomnography (PSG)-derived variables, using a TDI score threshold ≤ 30 as the dependent variable. Factors exhibiting *p*-values < 0.1 entered multivariate logistic regression analysis to pinpoint independent predictors associated with olfactory dysfunction. Potential multicollinearity was evaluated by calculating the variance inflation factor (VIF), with variables demonstrating a VIF greater than 5 excluded to prevent collinearity bias. Adjusted odds ratios (ORs) and 95% confidence intervals (CIs) were derived through forward stepwise regression. The statistical significance level was predetermined at *α* = 0.05. Sensitivity analysis stratified by OSAHS severity (mild: AHI 5–15; moderate: 15–30; severe: >30 events/h) was performed.

## 3 Results

### 3.1 General information

Based on TDI total scores, patients were categorized into two groups: the olfactory dysfunction group (112 individuals) and the normal olfaction group (86 individuals). Analysis revealed a significantly greater proportion of male patients and older age within the olfactory impairment group compared to those without impairment (*p* < 0.05). Additionally, the olfactory dysfunction group exhibited lower TST, SE, REM-stage microarousals, NREM-stage microarousals, the ratio of stage N3 to TST (N3/TST%), and the ratio of REM to TST (REM/TST%) than those with normal olfaction (*p* < 0.05). Conversely, AHI and WASO values were significantly elevated in the olfactory dysfunction group (*p* < 0.05). There was no statistically significant difference in years of education, the longest duration of OA, the lowest oxygen saturation, N1/TST%, and N2/TST% between the two groups (*p* > 0.05) (Table 1). Smoking, alcohol use, and diabetes showed no significant association with TDI (*p* > 0.05).

**Table 1:** Comparison of general information.

Group	The normal olfactory group	Olfactory disorder group	*χ*^2^/*t*/*z*	*P*
Gender/example			4.370	<0.05
Male	50	81		
Female	36	31		
Age/years	40.48 ± 5.32	55.79 ± 5.90	−18.89	<0.05
Years of education/years	9.0(8.0,11.0)	9.0(8.0,10.5)	−0.230	0.818
PSG				
The maximum duration of OA/min	55.62 ± 7.24	54.66 ± 6.44	0.980	0.328
TST/min	411.86 ± 50.98	356.29 ± 51.99	7.517	<0.05
Minimum oxygen saturation/%	79.85 ± 4.78	79.33 ± 5.47	0.704	0.482
SE/(%)	88.27 ± 15.88	73.99 ± 14.54	6.580	<0.05
WASO/min	41.83 ± 14.24	50.93 ± 13.53	−4.587	<0.05
Total number of micro-awakenings during REM sleep	22(14,29)	13.5(6,19)	−5.612	<0.05
The total number of micro-awakenings during NREM sleep stage	171(151,188)	106(84,134)	*z* = −6.905	<0.05
N1/TST%/%	30.51 ± 4.00	30.41 ± 3.64	*t* = 0.188	0.851
N2/TST%/%	45.25 ± 5.24	44.75 ± 4.83	*t* = 0.701	0.484
N3/TST%/%	6.71(5.72,8.25)	4.73(3.63,6.3)	*z* = −5.870	<0.05
REM/TST%/%	15.52(12.25,18.1)	9.61(7.7,13.21)	*z* = −7.045	<0.05
AHI/(times/h)	18.41 ± 4.83	27.93 ± 5.13	*t* = −13.279	<0.05
TDI score				
Total (TDI)	36(34,40)	25(22,27)	*z* = −12.065	<0.05
Threshold (T)	13(11,15)	9(8,11)	*z* = −7.492	<0.05
Discrimination (D)	12(10,14)	8(6,10)	*z* = −8.355	<0.05
Identification (I)	11.81 ± 2.83	7.04 ± 2.56	*t* = 12.422	<0.05

### 3.2 Correlation analysis of olfactory function and sleep structure parameters in OSAHS patients

**Figure 1:**
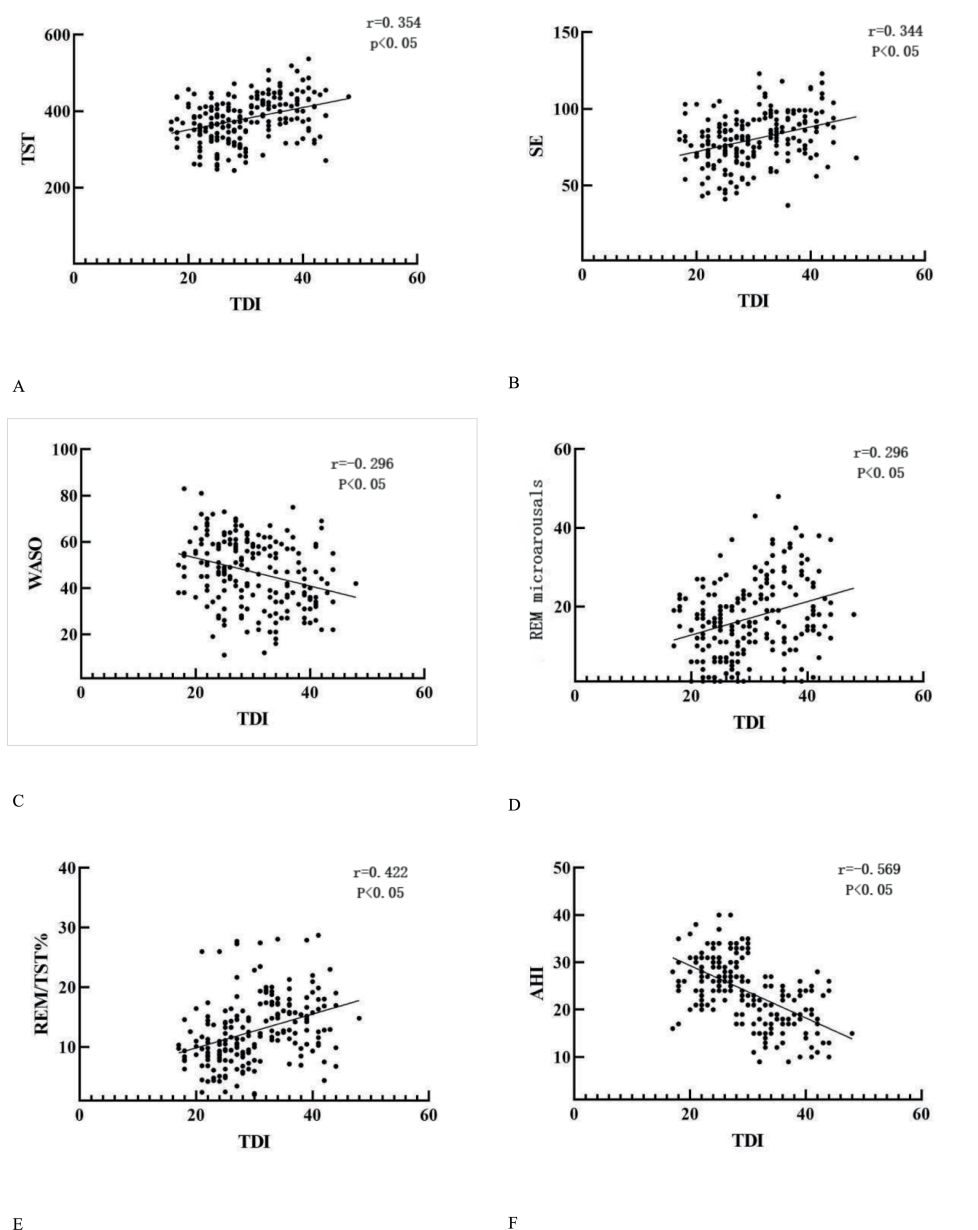
Correlation analysis of olfactory function and sleep structure parameters in OSAHS patients. **(A)** Scatter plot of the correlation between the total TDI score and TST. **(B)** Scatter plot of the correlation between the total TDI score and SE. **(C)** Scatter plot of the correlation between the total TDI score and WASO. **(D)** Scatter plot of the correlation between the total TDI score and the total number of microarousals in REM. **(E)** Scatter plot of the correlation between the total TDI score and REM/TST%. **(F)** Scatter plot of the correlation between the total TDI score and AHI.

Correlation analyses utilizing Pearson and Spearman methods indicated significant negative correlations between the total TDI score and age (*r* = −0.697, *p* < 0.05), AHI (*r* = −0.569, *p* < 0.05), and WASO (*r* = −0.296, *p* < 0.05). Conversely, TDI scores were positively correlated with TST (*r* = 0.354, *p* < 0.05), SE (*r* = 0.344, *p* < 0.05), the total count of microarousals during the REM stage (*r* = 0.296, *p* < 0.05), and REM/TST% (*r* = 0.422, *p* < 0.05). A weak positive association was observed between total TDI score and the proportion of N3 sleep (N3/TST%). However, no statistically significant relationships were found between TDI scores and gender, educational duration, maximum duration of obstructive apnea (OA), minimum oxygen saturation, total microarousals during NREM sleep, and the proportions of N1 and N2 sleep stages (N1/TST%, N2/TST%) (*p* > 0.05) (Table 2 and Figure 1).

**Table 2:** Correlation analysis of olfactory function and sleep structure parameters in OSAHS patients.

Variable	TDI	
	*r*	*P*
Gender	0.138^①^	0.052
Age	−0.697^②^	<0.05
Education years	−0.107^①^	0.133
The maximum duration of OA	0.063^②^	0.379
TST	0.354^②^	<0.05
Minimum oxygen saturation	0.090^②^	0.205
SE	0.344^②^	<0.05
WASO	−0.296^②^	<0.05
Total number of micro-awakenings during REM sleep	0.296^①^	<0.05
The total number of micro-awakenings during NREM sleep stage	0.081^①^	0.259
N1/TST%	0.053^②^	0.462
N2/TST%	−0.010^②^	0.891
N3/TST%	0.169^①^	<0.05
REM/TST%	0.422^①^	<0.05
AHI	−0.569^②^	<0.05

① indicates the use of Spearman correlation test, ② indicates the use of Pearson correlation test.

### 3.3 Multivariate logistic regression analysis

Taking the total score of TDI as the dependent variable and age, AHI, WASO, TST, SE, the total number of microarousals during REM sleep, and REM/TST% as independent variables, and adjusting for gender, the VIF analysis showed that WASO and the total number of microarousals during REM sleep were collinear (VIF > 5) and did not enter the final model (*p* > 0.05). The results of the multivariate logistic regression analysis indicated that age, AHI, and REM/TST% were independent influencing factors of TDI, as shown in Table 3. For each additional year of age, the risk of olfactory dysfunction increased by 83.8% (OR = 1.838). For each additional 1 time/h of AHI, the risk of olfactory dysfunction increased by 81.1% (OR = 1.811). For each 1% increase in the proportion of REM sleep (REM/TST%), the risk of olfactory dysfunction decreased by 25.5% (OR = 0.745).

**Table 3:** Multivariate logistic regression analysis of the total score of TDI.

Variable	*β*	S. E.	Wald	*P*	OR	95%CI	
						Lower limit	Upper limit
Age	0.609	0.143	18.109	<0.05	1.838	1.389	2.433
REM/TST%	−0.294	0.112	6.913	<0.05	0.745	0.599	0.928
AHI	0.594	0.172	11.983	<0.05	1.811	1.294	2.535
Quantity	−38.603	9.087	18.048	<0.05	–	–	–

Correct for gender factors.

## 4 Discussion

Obstructive sleep apnea-hypopnea syndrome, a systemic disease characterized by sleep structure disorder and intermittent hypoxia, has drawn increasing attention for its multi-system damage. A multicenter nomogram prediction model study indicated that a nocturnal oxygen saturation below 90% as detected by PSG is a risk factor for olfactory dysfunction in OSAHS patients (10). However, contrary to the emphasis on hypoxic burden in previous models, our results revealed a discordance: no significant correlation was observed between static hypoxia markers (such as the lowest blood oxygen saturation, LSaO₂) and TDI scores (*r* = 0.090, *p* = 0.205). Instead, changes in REM sleep architecture showed a stronger association (*r* = 0.422, *p* < 0.001). This discordance may arise because LSaO₂ merely represents a single nadir point during sleep, failing to reflect the cumulative dynamic fluctuations of oxygenation and the specific vulnerability of neural circuits during different sleep stages. REM sleep is a critical stage for synaptic plasticity and the consolidation of olfactory memory in the piriform cortex and hippocampus (6, 11). Therefore, while chronic intermittent hypoxia (measured by LSaO₂) may contribute to generalized neuronal damage, the specific reduction in REM sleep appears to be a more sensitive indicator of functional olfactory impairment in OSAHS.

The results of this study demonstrated that AHI and REM/TST% are independent influencing factors for olfactory dysfunction. Specifically, for every 1% increase in REM sleep proportion in OSAHS patients, the risk of olfactory dysfunction decreases by 25.5%, and for every 1 increase in AHI per hour, the risk of olfactory dysfunction increases by 81.1%. Previous studies have shown that olfactory dysfunction in OSAHS patients involves multi-system pathophysiological changes, including: ① nasal airflow limitation preventing odor molecules from effectively reaching the olfactory mucosa’s olfactory neurons, blocking the physical conduction of olfactory signals; ② intermittent hypoxia in OSAHS patients leads to elevated levels of serum inflammatory factors (such as IL-6, IL-17), inducing local inflammatory responses in the olfactory mucosa and olfactory bulb, and damaging olfactory neurons; ③ chronic intermittent hypoxia in OSAHS patients can trigger oxidative stress, mitochondrial dysfunction, and neuronal apoptosis, and olfactory dysfunction may be an early marker of neuronal apoptosis (12–16). A high AHI indicates more frequent apnea events and a decrease in blood oxygen saturation. OSAHS patients experience sleep disruption, intermittent hypoxemia, and a decline in REM sleep quality. REM sleep is a critical stage for odor memory consolidation (11). A decrease in REM/TST% leads to a decline in synaptic plasticity in the olfactory cortex (such as the piriform cortex and hippocampus), affecting odor recognition and cognitive function. All OSAHS patients included in this study were excluded from factors causing nasal airflow limitation, such as nasal surgery history, chronic sinusitis, and acute exacerbation of allergic rhinitis. Therefore, it is speculated that the olfactory dysfunction in OSAHS patients in this study may be related to abnormal sleep structure and autonomic nerve dysfunction.

Prior research has identified gender as an independent determinant of olfactory dysfunction in OSAHS patients, noting a decline in olfactory performance associated with advancing age, and generally better olfactory threshold, discrimination, and recognition scores among females compared to males (10, 17, 18). Consistent with these findings, this study revealed higher proportions of male patients and a greater mean age in the olfactory impairment group relative to those with normal olfactory function. Gender-based differences in olfactory capability may be attributed to hormonal variations across sexes (19). Additionally, other research has indicated that asymmetry in the superior temporal gyrus, a region intimately involved in olfactory processing, is more pronounced in males compared to females, potentially contributing to observed gender differences in olfactory function (20). Although gender was adjusted for in the regression models, the male predominance in the sample may still introduce selection bias. Moreover, this study did not further analyze nasal resistance or olfactory mucosal imaging data of the patients; future research should incorporate nasal resistance measurements and imaging examinations to explore the impact of gender-specific nasal structural differences on olfactory function in OSAHS patients. Second, the absence of a concurrent healthy control group limits the generalizability of our findings; however, the strict exclusion criteria and within-group stratified analysis effectively strengthen the inferences regarding OSAHS-specific pathological mechanisms. Furthermore, the cross-sectional design itself precludes causal inference between sleep architecture and olfactory dysfunction, necessitating future prospective cohort studies involving continuous positive airway pressure (CPAP) interventions to validate these findings.

This study observed a strong association between age and olfactory dysfunction (OR = 1.838), and the TDI total score showed a nonlinear decline trend with age (*R*^2^ = 0.47). This age effect may be related to the following mechanisms: ① the density of olfactory receptor neurons in the olfactory epithelium decreases at a rate of 0.3% per year with age (21); ② atrophy of the orbitofrontal cortex volume leads to a decline in advanced olfactory processing ability (22); ③ elderly patients have a more significant degree of REM sleep fragmentation. Notably, although age has a significant impact on olfactory function, the predictive value of REM/TST% remains independent of age (adjusted *β* = −0.294), suggesting that sleep structure parameters may be more sensitive biomarkers than age. Although this study confirmed the predictive value of REM/TST%, there are still the following limitations: ① The cross-sectional design cannot determine the causal relationship, and a prospective study with CPAP intervention is needed to verify the temporal association between sleep structure improvement and olfactory recovery; ② Serum IL-6, TNF-*α* and other inflammatory factors were not detected, making it difficult to quantify the role of systemic inflammation in the “sleep structure disorder-olfactory impairment” axis; ③ Olfactory testing only used Sniffin’ Sticks, and future studies need to combine functional magnetic resonance imaging (fMRI) to observe changes in the activation pattern of the olfactory cortex. Future research directions include: ① Establishing a dynamic monitoring database of olfactory function in OSAHS patients, and using multimodal imaging (such as PET-MRI) to track the association between the volume of the olfactory bulb and sleep parameters; ② Conducting randomized controlled trials to compare the effects of different treatment methods such as continuous positive airway pressure (CPAP) and mandibular advancement devices (MAD) on REM sleep structure and olfactory function; ③ Exploring the application potential of REM sleep modulators (such as orexin receptor antagonists) in olfactory protection. This study provides new ideas for individualized management of OSAHS patients: ① It is recommended to incorporate REM/TST% into the OSAHS disease assessment system, especially in high-risk populations with cognitive impairment; ② For patients with REM sleep proportion <15%, olfactory function screening should be strengthened and active intervention measures should be prioritized; ③ Developing an olfactory disorder risk prediction model based on sleep structure parameters (such as Nomogram) to enhance early warning capabilities.

## 5 Conclusion

Age increase, elevated AHI, and decreased REM/TST% are independent influencing factors for the occurrence of olfactory disorders. The decline in olfactory function is significantly associated with a marked reduction in REM sleep. Elderly patients with OSAHS, high AHI, and low REM proportion should be the key population for olfactory function screening.

## Data Availability

The original contributions presented in the study are included in the article/supplementary material, further inquiries can be directed to the corresponding author.
